# The Telephote

**Published:** 1914-10

**Authors:** 


### The Telephote.

This instrument consists of a receiving disk saturated with various chemicals having a special selective affinity for the heat, light and actinic rays emanating from any source of illumination. The impact of these rays—and we need not discuss here whether they consist of vibrations or of minute particles or of both—produces minute differences in electric tension which are transmitted by the familiar process of relays as electric currents of appreciable amperage to an analogous transmitter, in which essentially the reverse of the phenomena at the transmitter takes place. From the transmitter, therefore, rays of light in the strict sense, as well as associated heat and actinic rays are given off so as, in a crude practical sense, to reproduce the original light at a distance—whence the name of the instrument.

It can readily be understood that by limiting the materials saturating the receiving disk, any one or two or the entire three sets of rays may be reproduced at a distance. It can also be appreciated that purely technical difficulties prevent the absolutely accurate reproduction, at the transmitter of the original rays in their full strength and proportions. Thus, save for purely scientific purposes, it is impracticable to transmit to a distance ordinary artificial light of feeble intensity, as, for instance, that from a candle, kerosene lamp, gas jet, with or without incandescent mantel or electric bulb. For commercial and industrial purposes, this limitation is not of great importance. Neither, except for purely scientific investigation, is there a demand for the transmission of the light of volcanic eruptions, of the moon, planets or stars.

Thus, the practical value of the telephote is limited to the transmission of sun light, on account of its intensity, general availability, ubiquity and cheapness. As the sun, with reference to any terrestrial point, is a movable body, due to a combination of terrestrial revolution, orbital progress and other factors well known to astronomers, the receiving apparatus of any telephote—or in this limited sense, teleheliophote—must necessarily be adjusted upon a revolving apparatus, driven by clock work or similar device, so as always to present a surface nearly or quite at right angles to the direction of the rays at that point. Under certain conditions, economy is also served by concentrating mirrors, under which circumstances, it is more convenient to alter the angle of the mirrors, while the actual receiver is stationary. For other purposes, as the lighting of auditoriums, study rooms, places of business, stores, etc., an automatic arrangement of shades to maintain a steady, moderate supply of light, is more convenient and even more economic than similar simple devices at the transmitter although these latter involve no more trouble than the use of shades at windows to which we have long been accustomed.

In its rudimentary, short distance, wire-conducted form, the teleheliophote is obviously limited to the duration of the day light at any particular time and place and the transmission of bright sunlight is, of course, limited by clouds. If this general principle can be extended to long distance, especially if some wireless means of transmission can be perfected, it is obvious that we shall be entirely independent of the length of the day and, since one or another station may be used for transmission, of local climatic conditions. Thus, with this extension, artificial lighting, for interiors, streets and roads, even for the stimulation of vegetable growth, will be entirely under human control and it is not inconceivable that climate may be modified especially by the selective transmission of heat waves.

Even in the ordinary, short distance, wire-conducted form, the teleheliophote solves many problems of economics and hygiene. It is a general axiom that under normal conditions, including the absence of high degrees of commercial exploitation, the cost of an article or power depends mainly on the cost of the original production and not of transportation. In this instance, the source of light is free and present in supra-abundance in any space for which it is needed. While the instruments are delicate and expensive, the actual cost of transmission and management is slight.

For art galleries, museums, libraries, study rooms, factories, and, especially for the display of certain fabrics, natural day light is so far superior to any artificial substitute hitherto employed that it may be said to be cheap at any price; and this statement is true from the economic as well as the hygienic and aesthetic standpoint.

As is well known, sun light, especially bright and direct but also in diffused, reflected form, is a potent germicide. The hygienic advantage of absence of products of combustion and the safety of absence of high voltage electric currents, as well as the direct ocular hygienic factor involved need merely to be mentioned. The economic advantage of a source of light independent of external exposure will greatly simplify the construction of buildings, especially in crowded parts of cities and will reduce the cost of lodging to the poor without sacrifice of sanitary demands. Even in buildings in which this problem is not so acute, the ability to direct sun light into all portions will undoubtedly greatly reduce the fomites of various bacteria, notably of tuberculosis.

The only trouble about the telephote is that it has not yet been invented. We suggest this problem to inventors as one of the widest humanitarian value, not to mention the enormous commercial value.

This was written in the spring of 1914. We clip the following from Popular Electricity and Modern Mechanics, September, 1914:

''Seeing by Wire—A London engineer claims to have discovered a method by which light may be transmitted by wire. The apparatus consists of a screen transmitter divided into a number of small squares, each being a cell of selenium, the electrical resistance of which varies according to the strength of light falling upon it. Over the screen is passed a running roller consisting of a number of pieces which are alternately conductors and insulators. The roller is driven by a motor at 300 revolutions per minute, and the resulting variations of light are transmitted along the conducting wire. The receiver is made up of a series of cells operated by the passage of polarized light through thin slats of steel and the object before the transmitter is produced at the receiver as a flickering image. The system has been successfully tested for distances up to four miles. It is stated that the selenium may be replaced by any dimagnetic material.

